# Characterizing bracken fern phenological cycle using time series data derived from Sentinel-2 satellite sensor

**DOI:** 10.1371/journal.pone.0257196

**Published:** 2021-10-28

**Authors:** Trylee Nyasha Matongera, Onisimo Mutanga, Mbulisi Sibanda

**Affiliations:** 1 Discipline of Geography, University of KwaZulu-Natal, Scottsville, Pietermaritzburg, South Africa; 2 Department of Geography, Environmental Studies and Tourism, University of Western Cape, Cape Town, South Africa; Potsdam Institute for Climate Impact Research, GERMANY

## Abstract

Bracken fern is an invasive plant that has caused serious disturbances in many ecosystems due to its ability to encroach into new areas swiftly. Adequate knowledge of the phenological cycle of bracken fern is required to serve as an important tool in formulating management plans to control the spread of the fern. This study aimed to characterize the phenological cycle of bracken fern using NDVI and EVI2 time series data derived from Sentinel-2 sensor. The TIMESAT program was used for removing low quality data values, model fitting and for extracting bracken fern phenological metrics. The Sentinel-2 satellite-derived phenological metrics were compared with the corresponding bracken fern phenological events observed on the ground. Findings from our study revealed that bracken fern phenological metrics estimated from satellite data were in close agreement with ground observed phenological events with R^2^ values ranging from 0.53–0.85 (*p < 0*.*05*). Although they are comparable, our study shows that NDVI and EVI2 differ in their ability to track the phenological cycle of bracken fern. Overall, EVI2 performed better in estimating bracken fern phenological metrics as it related more to ground observed phenological events compared to NDVI. The key phenological metrics extracted in this study are critical for improving the precision in the controlling of the spread of bracken fern as well as in implementing active protection strategies against the invasion of highly susceptible rangelands.

## 1. Introduction

The encroachment of invasive species in productive rangelands influences changes in nutrient cycles [1], fire incidences and severity [2] and alters the abundance of biodiversity [3], resulting in socio-economic implications on livelihoods. Bracken (*Pteridium Aqulinimun*) is one of the most problematic alien invasive ferns that encroaches into new landscapes [4]. In South Africa, there is evidence of bracken fern encroachment in the Drakensberg Mountains. Although it is not clear how bracken fern was introduced in the Drakensberg, Finch, Hill [5] noted that archeological evidence suggests that the existence of bracken fern in the Drakensberg montane grasslands can be traced back to as far as 1840 CE. Due to its vigorous growth and dense canopy, the fern has negative impacts on agricultural productivity [6], animal and human health [7], forestry and recreational potential [8], leading to huge economic losses. Generally, farmers abandon the agricultural land once the fern heavily invades the land due to its persistent underground root system, which facilitates fast growth. The invasive fern invades grasslands and grazing pastures [9–11], while it also perseveres in woodlands and hedgerows, making it difficult for indigenous grass species to thrive [12]. Furthermore, the encroachment of bracken fern causes the reduction of herbaceous native vegetation and destruction of habitat in rangelands. The biochemical chemistry and morphology of bracken fern influence its spectral reflectance behavior. Specifically, bracken fern has various pigments and carotenoids pigments that forms part of the compound arrangement of the fern’s cells which actively absorb and distribute radiation and different wavelengths [13].

Understanding the biological structure and timing of bracken fern phenology improves rangeland management’s knowledge and ability to choose the suitable treatment method in areas infested by the fern [14]. The phenological information can be used to implement rapid response initiatives for the successful restoration of landscapes at different scales [14]. In literature, the prediction of future invasions before their occurrence has been postulated as one of the most efficient strategies of managing rangelands [15–17]. Therefore, understanding bracken fern phenology can help to predict how the fern species populations will change in time, and necessary proactive measures can be taken accordingly. Accurate and effective strategies in invasive species management save time and resources [18, 19]. A well-documented phenological cycle of bracken fern will assist conservationists and farmers in determining the most effective methods and appropriate time for controlling the fern across its life cycle stages, to ensure the complete eradication of the fern with minimum costs and inputs. For instance, knowing the beginning of the bracken fern season will help the rangeland managers with planning and implementing the appropriate control measures at an early phenological stage before the spores have been dispersed. Furthermore, the information on bracken fern’s phenology is vital in understanding the major drivers of its population dynamics and patterns of invasion. In this regard, an understanding of bracken fern’s phenological cycle will provide spatial information on areas that are more threatened for informing policy decisions on deriving effective control and management strategies. Over the past decades, remote sensing has proved to be an invaluable data source suitable for characterizing the phenological profile of vegetation at the local, regional and global scale [20–22]. Therefore, the uncontrolled colonization of bracken fern in the Drakensberg [23], ascertains the necessity to characterize its phenological cycle.

Earlier works on bracken fern phenology have used field-based studies [24, 25] and Phenology Cameras (PhenoCams) [26] to understand the phenological cycles of the fern in different parts of the world. However, the major limitation of these locally based methods is the spatial extent to which the plant phenological events were collected [27]. Remote sensing technology offers better prospects in providing archives of long term spatial data required to understand the phenological cycles of bracken fern at various scales. The use of remotely sensed data sets in retrieving the phenological metrics of vegetation is referred to as Land Surface Phenology (LSP) in remote sensing literature [28]. The application of remotely sensed data in estimating and monitoring LSP was pioneered by early satellite sensors such as Landsat series [29], Advanced Very High Resolution Radiometer (AVHRR) [30], the Moderate Resolution Imaging Spectroradiometer (MODIS) [31]. The AVHRR and MODIS satellite sensors have a high temporal resolution and synoptic views which is appropriate for large-scale monitoring of land surface phenology [30, 32, 33]. However, despite pioneering land surface phenology studies, the application of low spatial resolution sensors like AVHRR and MODIS is limited by their spatial resolution, calibration errors and poor geometric registration while Landsat is limited by its low temporal resolution. The freely available Sentinel-2 Multi-Spectral Instrument (MSI) optical sensor is composed of two satellites; Sentinel-2A and 2B, hence its revisit time has been decreased from 10 to 3–5 days. The sensor has improved sensor calibration with 10 – 60m spatial resolution which presents a potential for successful characterization of the phenological cycles of vegetation at the species level.

LSP scientists have utilized numerous spectral vegetation indices derived from satellite data to estimate the phenological cycles of vegetation at various scales [34–38]. Over the past decades, vegetation indices were developed and used as indicators of changes in vegetation structure, density, spatial extent and phenological timings. The Normalized Difference Vegetation Index (NDVI) and the Enhanced Vegetation Index (EVI2) have been commonly used to quantify the cyclical patterns of vegetation in different ecosystems [39–43]. The NDVI is a commonly used vegetation index regarded as a proxy indicator of vegetation canopy function and is directly associated with the absorption of photosynthetically active radiation by plant canopies while EVI2 was developed to enhance the vegetation signal with better sensitivity in areas with high biomass [44].

The characterization of the phenological profile of specific vegetation species using satellite data has mainly been done for crops [45–48], whilst the estimation of the phenological cycles of specific invasive species such as bracken fern still requires more attention. To the best of our knowledge there are no published studies that have used porlar orbiting satellite data sets such as Sentinel-2 to extract the phenological metrics of bracken fern for the purpose of improving its management approaches. Therefore, the first objective of this study was to characterize the phenological cycle of bracken fern using NDVI and EVI2 time series data derived from the Sentinel-2 satellite sensor. Secondly, the study sought to investigate the differences and similarities between NDVI and EVI2 data in estimating bracken fern phenological metrics. Finally, the study assessed the relationship between phenological metrics estimated from satellite data and the bracken fern phenological events recorded on the validation site, using Cathedral Peak World Heritage Site in the Drakensberg as the study site. This study is part of a continuing effort to craft an integrated approach to control the spread of invasive species in KwaZulu-Natal Nature reserves in South Africa.

## 2. Methods and materials

### 2.1 Study site description

The Cathedral Peak area is a World Heritage Site which falls within South Africa’s summer rainfall region. The Drakensberg climate is a result of a combination of factors such as altitude, topography as well as the Agulhas current in conjunction with atmospheric pressure system patterns over and adjacent to South Africa [49]. The mean annual rainfall in the Drakensberg is approximately 1800mm [50]. The maximum daily temperatures exceed 25 degrees Celsius while the minimum daily temperatures may drop below 0 degrees Celsius during winter [51]. The area is characterized by a mountainous environment that is largely dominated by C_3_ and C_4_ grass species such as Festuca and Themeda respectively [52]. The Cathedral Peak landscape is also characterized by invasive species that are increasingly encroaching into the grasslands. Nearly seventy-five categories of alien invasive plants have been recorded to exist in Drakensberg [53]. Bracken fern is amongst the commonly found invasive species that have been invading rangelands in the Drakensberg.

### 2.2 Ground observed phenology data

**Fig 1:**
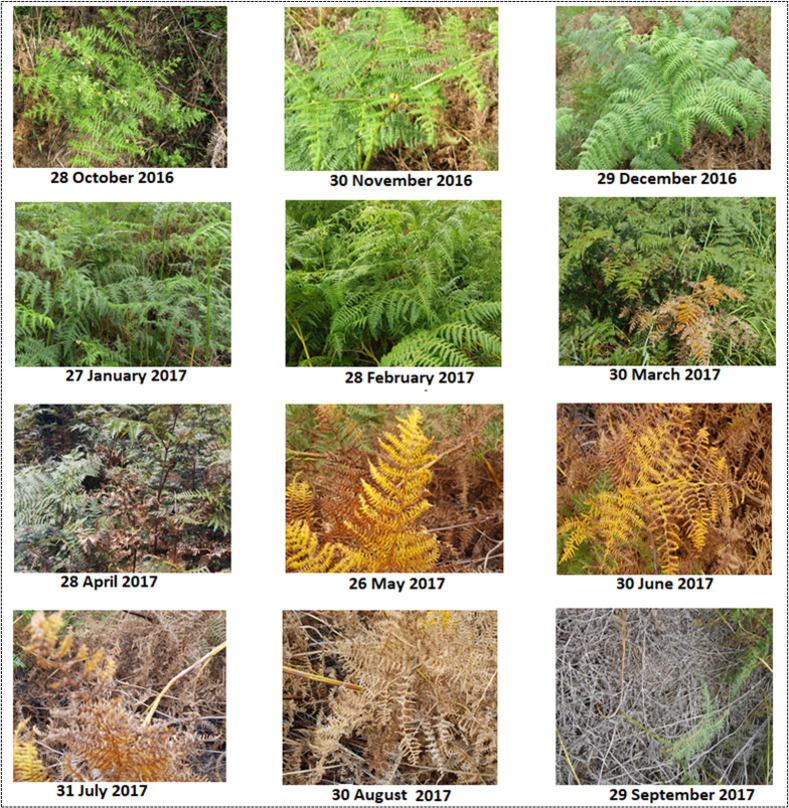
Phenological transformation of bracken fern appearance from October 2016 to September 2017 captured in Cathedral Peak study site.

The ground phenology recordings included the collection of bracken fern patches locations using a portable Leica GS20 Global Positioning System. A total of 60 bracken fern patches were collected. The bracken fern patches that were recorded were larger than 10 by 10 m (100m^2^) for them to match the Sentinel-2-pixel size as well as to account for geolocation errors of the GPS and the Sentinel-2 imagery. Purposive sampling was used to select bracken fern patches with more than 75% bracken percentage cover. The bracken fern phenological developments were recorded weekly from 1 January 2016 to 31 December 2018. Ferns like bracken develop fronds instead of leaves, for ground phenology observations in this study, the term ’fronds’ was used instead of leaves. Specifically, we recorded the dates of bracken fern frond emergence, expanded frond growth, withering and frond drying and considered them as green up, maturity, senescence and dormancy respectively. The recording of bracken phenological events on the ground was done with the assistance of the Department of Invasive Species for the Ezemvelo KwaZulu-Natal (KZN) Wildlife under the project lisence number 144/2017b. There were no plants and animals that were destroyed or killed during then data collection process. Fig 1 shows the phenological transformation of bracken fern appearance from October 2016 to September 2017. The bracken fern photographs were captured weekly, but only monthly images were shown since they proved to be sufficient to show the change in the phenological appearance of the fern. For consistency, the photographs were captured in the same position, at a bracken fern patch located at 29°13’26.758"E 28°56’46.2"S.

### 2.3 Satellite data acquisition and pre-processing

Sentinel-2 Multispectral Instrument (MSI) satellite images were obtained from the European Space Agency (ESA) online platform (https://earthexplorer.usgs.gov/). Sentinel-2A and 2B satellite images were included in the time series data for bracken fern phenological analysis. The satellite images were acquired at the processing level 1C. The images were atmospherically and geometrically corrected by ESA. A total of 108 images from January 2016 to December 2018 were acquired. Sentinel-2 images with less than 20% cloud cover were selected and included in the phenological analysis. The Function of mask (Fmask) 4.0 algorithm was used for detecting and removing clouds and shadows in the satellite images. For more details about the Fmask 4.0 algorithm procedure, see Qiu, Zhu [54].

The Normalized Difference Vegetation Index (NDVI) [55] and the two band Enhanced Vegetation Index (EVI2) [56] were used to extract the bracken fern phenological metrics. The NDVI was chosen based on its long term successfully applications in the phenology studies [57–59]. NDVI is suitable for both local and large-scale vegetation assessments, related to canopy structure and canopy photosynthesis, an attribute that is very crucial in the current study. EVI2 was also used based on its sensitivity to coherent inter-band (blue, red and NIR) atmospheric correction and thus may become much better over extreme bright or dark surfaces, such as subpixel clouds, desert playas, and inland water bodies, where the EVI values are usually problematic [60]. Additionally, EVI2 has also been reported to solve resolve Leaf area Index (LAI) differences for vegetation with different background soil reflectance [61]. The NDVI and EVI2 indices were calculated using the 108 Sentinel-2A and 2B satellite images in the TerrSet Geospatial Monitoring and Modelling System (Version 18.21) software based on the following equations:

NDVI=(NIR-RED)/(NIR+RED) (Eq 1)

EVI2=G(NIR−Red)/(NIR+(6−7.5/C)Red+1) (Eq 2)

where NIR, red and blue represents the quantity of NIR, red and blue light reflected by vegetation and measured by the satellite sensor [62], 2.5 is the gain or scaling factor; 6 and 7.5 are coefficients of the aerosol resistance term while 1 represents the canopy background adjustment for correcting the nonlinear, differential NIR and red radiant transfer through a canopy. G will be determined in accordance with the c value. The NDVI and EVI2 images were exported to TIMESAT program for further analysis.

### 2.4 Data smoothing and phenological metrics extraction

**Fig 2:**
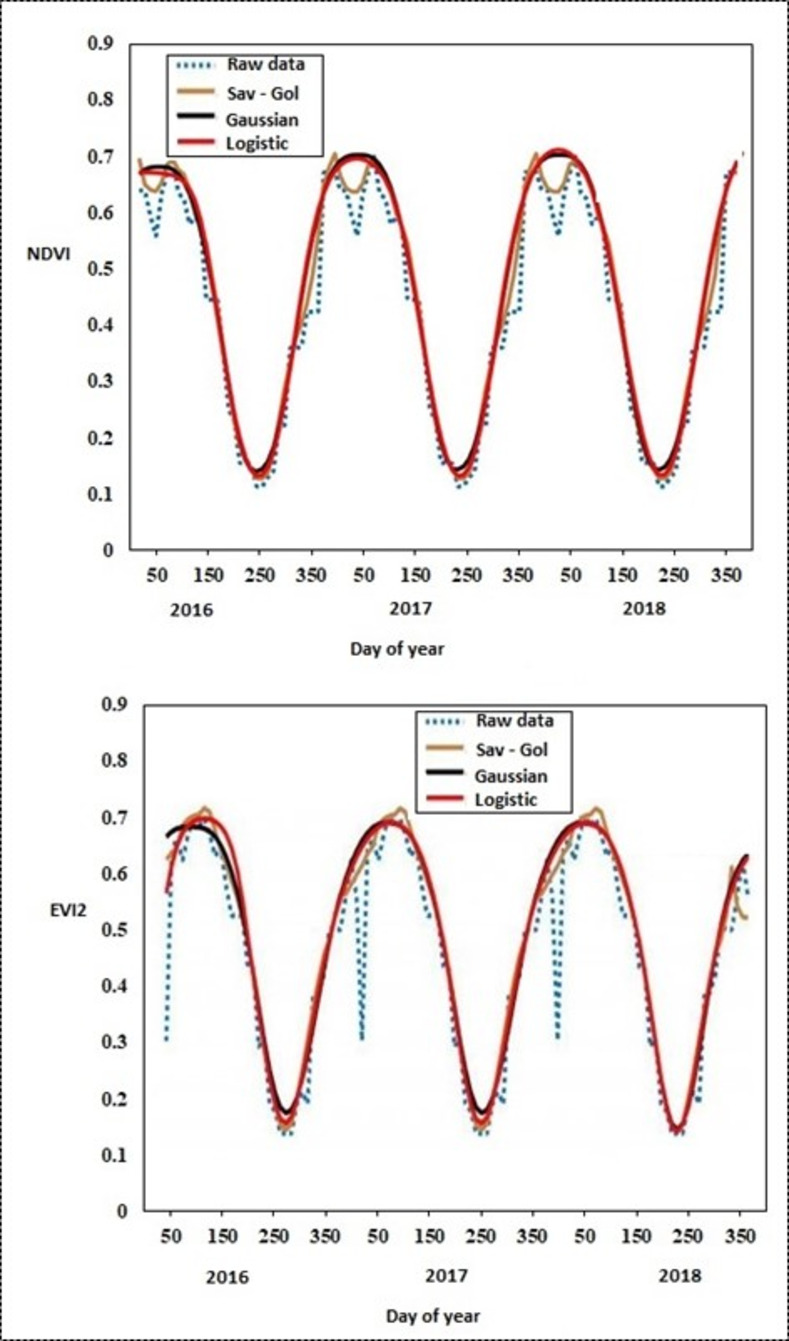
Graphical representation of (a) NDVI and (b) EVI2 raw and fitted time series data computed using SG, DL and AG models for a bracken fern pixel located in Cathedral Peak (29°13’26.758"E 28°56’46.2"S).

The current study used TIMESAT 3.3 program for processing vegetation indices time series data and estimating bracken fern phenological metrics. The TIMESAT program provides an understandable Matlab based user interface which facilitates the manipulation of data into vegetation phenological parameters. Specifically, three main processing stages were executed in TIMESAT: (1) preprocessing of NDVI and EVI2 time series data by detecting and removing outliers, (2) data smoothing and gap filling using the SG, DL and AG models based on the procedures which are described in detail by Eklundha and Jönsson [63] and (3) extraction of bracken fern phenological metrics. Fig 2A and 2B show the temporal trajectory of raw and smoothed NDVI and EVI2 time series respectively from 2016 to 2018 for a pixel located in the Cathedral Peak at co-ordinates (29°13’26.758"E 28°56’46.2"S). To provide the most robust description of bracken fern seasonal dynamics, 10-fold leave one out cross validation was used to automatically select the smoothing parameters for the SG, AG and DL smoothing functions as described in detail by Craven and Wahba [64].

In the current study, the bracken fern growth cycle was characterized by four main transition dates which are; 1) green up: indicating the date of onset of vegetation indices increase, 2) maturity: the date indicating the onset of vegetation indices maximum, 3) senescence: the date of onset of vegetation indices decrease and 4) dormancy: the date of onset of vegetation minimum. The TIMESAT program relies on the assumption that the growing seasons begin and end at a similar time annually. In principle, the start and end of the season for the targeted year is identified in the same time period as the first and third years [33]. The seasonal amplitude threshold method was used to extract bracken fern phenological metrics. The seasonal amplitude method is defined between the base level and the maximum value for each individual season [63]. The principle of the seasonal amplitude method states that the start of the season occurs when the left section of the fitted curve has reached a specified fraction of the amplitude, which is counted from the base level. The end of the season is also defined similarly but using the right side of the fitted curve. In this study, the start of the bracken fern season was defined as the day of the year when the vegetation indices surpassed 10% of the distance between the left minimum level and the maximum, while the end of the season is defined in a similar way, but in the opposite direction. For more details about the threshold fraction specifications, see the original technical paper by Jönsson and Eklundh [65].

### 2.5 Statistical analysis

**Fig 3:**
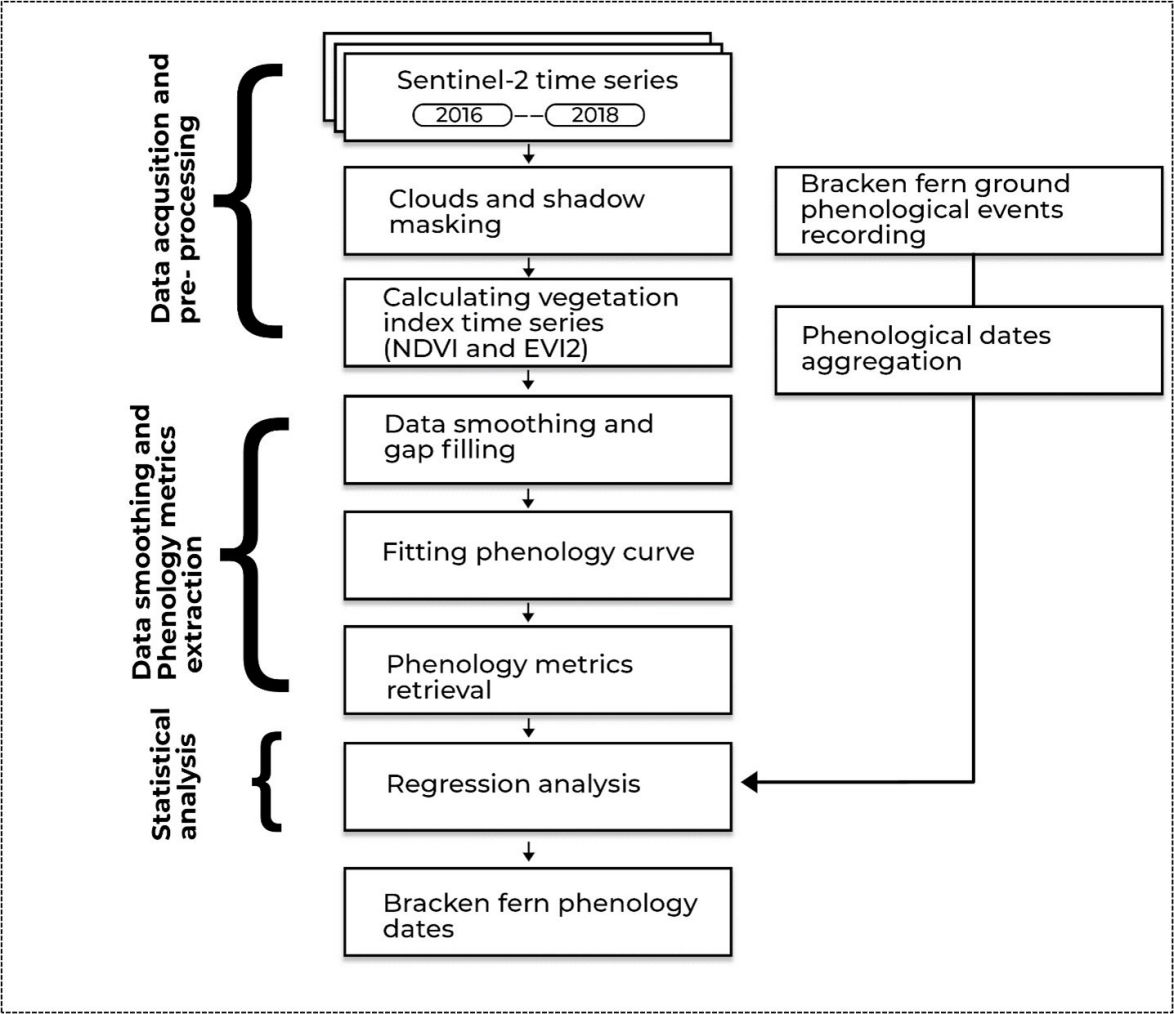
Schematic diagram illustrating the research methodology adopted in this study.

To assess the statistical relationships between satellite-derived phenological metrics and ground observed bracken fern phenological events, the coefficient of determination (R^2^) [66], the Root Mean Square Deviation (RMSD) [67] and the Mean Absolute Bias (MAB) [68] were computed. For comparison between satellite retrieved and ground observed phenological dates, the linear regression analysis was computed by using the ground observed phenological events as the independent variable and the satellite-derived vegetation indices as the dependent variable. The linear regression analysis was also conducted between NDVI and EVI2 phenological retrievals with NDVI and EVI2 retrievals as independent and dependent variables, respectively. The significance test for all the phenology models were conducted using the F-test with the standard 0.05 cut off indicating statistical significance between variables (P < 0.05). Fig 3 shows the flow chart illustrating the research methodology that was adopted in this study.

## 3. Results

### 3.1 Variation in TIMESAT models phenological retrievals

Table 1 shows a summary of bracken fern phenological metrics computed from the three models embedded in the TIMESAT program. Comparison of the mean phenological dates estimated using the three models revealed that bracken fern phenological dates from each model were different although their discrepancies were all less than 15 days. The statistical analysis revealed that the variance in the estimated phenological dates produced by the three models were statistically signficant (*p < 0*.*05)* for all the bracken fern phenological stages based on both NDVI and EVI2 time series. Results obtained using NDVI phenological retrievals shows that the mean bracken fern green up onset dates for the AG, SG and the DL were approximately around day 298, 296 and 294 while EVI2 dates were estimated to be around day 280, 288 and 284 respectively. Using the calendar dates, the average timing of bracken fern green up onset dates was towards the end of October 2016. The EVI2 green up onset dates were generally earlier than NDVI dates by an average of 11 days across the three models. For all the models, the standard deviations for the green up onset retrievals were consistent with a range of 2 to 5 days. Based on the NDVI phenological estimations, the DL model recorded the lowest deviation of 2.96 days while the SG NDVI model reported the highest deviation of 5.3 days.

**Table 1:** Mean phenological dates and standard deviations for the three models embedded in TIMESAT.

Model	Phenological metric	Mean (DOY)		Calendar Date	Standard Deviation (Days)	
		NDVI	EVI2		NDVI	EVI2
SG	GU	296	288	October; 2016	5.3	3.78
	MAT	57	53	February; 2017	3.36	3.04
	SEN	101	88	April; 2017	5.36	3.91
	DM	176	196	July; 2017	2.03	2.52
DL	GU	294	284	October; 2016	4.96	2.96
	MAT	56	54	February; 2017	3.86	2.56
	SEN	99	90	April; 2017	3.69	2.92
	DM	177	184	July; 2017	6.23	4.38
AG	GU	298	280	October;2016	3.76	4.73
	MAT	54	44	February;2017	6.11	7.35
	SEN	96	88	April;2017	3.34	2.57
	DM	181	190	July;2017	5.59	3.82

where GU = green up; MAT = maturity; SEN = senescence; DM = dormancy.

The NDVI maturity onset dates for the AG, SG and the DL were estimated to have occurred around day 54, 57 and 56 while the EVI2 dates were predicted to be around day 44, 53 and 54 respectively. Using the calendar dates, the estimated bracken fern maturity dates were towards the end of February 2017. The NDVI maturity onset dates were later than EVI2 dates by an average of 8 days across the three models. Compared to the green up the phenological stage, the maturity standard deviations were higher across all the models ranging from 2 to 7 days. The bracken fern green decrease was associated with the plummet in the vegetation index signal which signified the onset of the senescence phenological stage. Based on the AG, DL and SG the NDVI estimated date of senescence onset was around day 96, 99 and 101 while EVI2 retrievals estimated day 88, 90 and 81 respectively. The standard deviations ranged from 2 to 5 days across all models. The NDVI retrievals predicted the onset of dormancy stage to be around day 181, 177 and 176 for AG, DL and SG, while EVI2 retrievals were estimated to be around day 190, 184 and 196 respectively. For both NDVI and EVI2 phenological retrievals, the DL dormancy dates were earlier when compared to the other two models by an average of 16 days. The SG model had the lowest standard deviations (NDVI = 2.03 and EVI2 = 2.52), while the DL recorded the highest deviations (NDVI = 6.23 and EVI2 = 3.42).

### 3.2 Intercomparison of NDVI and EVI2 phenological retrievals

**Fig 4:**
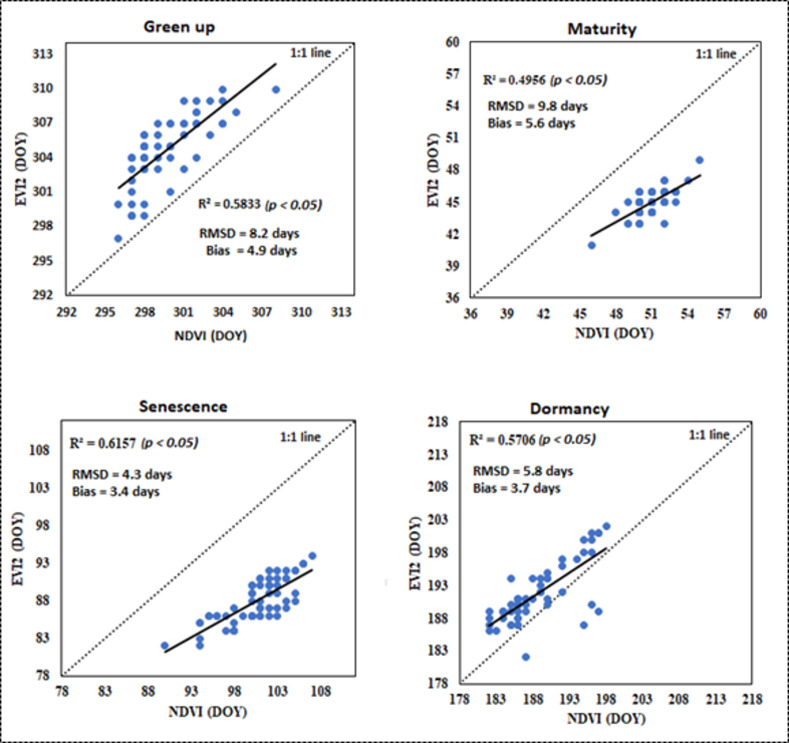
Statistical comparison between NDVI and EVI2 derived bracken fern phenological dates.

To compare bracken fern phenological metrics retrieved using NDVI and EVI2 time series, we averaged the dates of the four metrics estimated using the three models embedded in TIMESAT. Findings from our study demonstrated that the phenological metrics estimated using NDVI and EVI2 across the four major bracken fern phenological stages were comparable. The statistical analysis on Fig 4 shows scatter plots depicting the agreement between bracken phenological metrics estimated using the two vegetation indices. The EVI2 and NDVI phenological metrics show signficant linear relationships between each other amongst all phenological stages (*p < 0*.*05*) although the correlation coefficients were weak for some of the phenological stages with R^2^ values ranging from 0.49–0.61. For the green up onset stage, the coefficient of determination (R^2^ = 0.58) indicated a good correlation between NDVI and EVI2 phenological retrievals. The EVI2 phenological dates for green up onset were earlier than NDVI retrievals for most of the pixels across the study site. The green up onset RMSD was 8.2 days while a bias of 4.9 days was recorded.

The maturity stage recorded the lowest correlation between NDVI and EVI2 phenological retrievals (R^2^ = 0.49), the highest RMSD (9.8 days) as well as the highest bias of 5.6 days. Like the green up onset dates, the EVI2 retrievals recorded earlier green up onset dates compared to the NDVI retrievals. The senescence stage recorded a good correlation (R^2^ = 0.61) for the phenological retrievals between the two vegetation indices and a relatively moderate RMSD of 4.3 days and a bias of 3.4 days. The highest correlation between NDVI and EVI2 phenological retrievals was reported in the dormancy phenological stage (R^2 =^ 0.57) while the RMSD recorded 5.8 days and bias was 3.7 days. Generally, the agreement was moderate, and bias was low between phenological metrics estimated using the two vegetation indices across the four bracken fern phenological stages. The EVI2 phenological retrievals were mostly earlier than NDVI retrievals across the four bracken fern phenological stages.

### 3.3 Comparison between satellite-based phenological retrievals and ground observations

**Fig 5:**
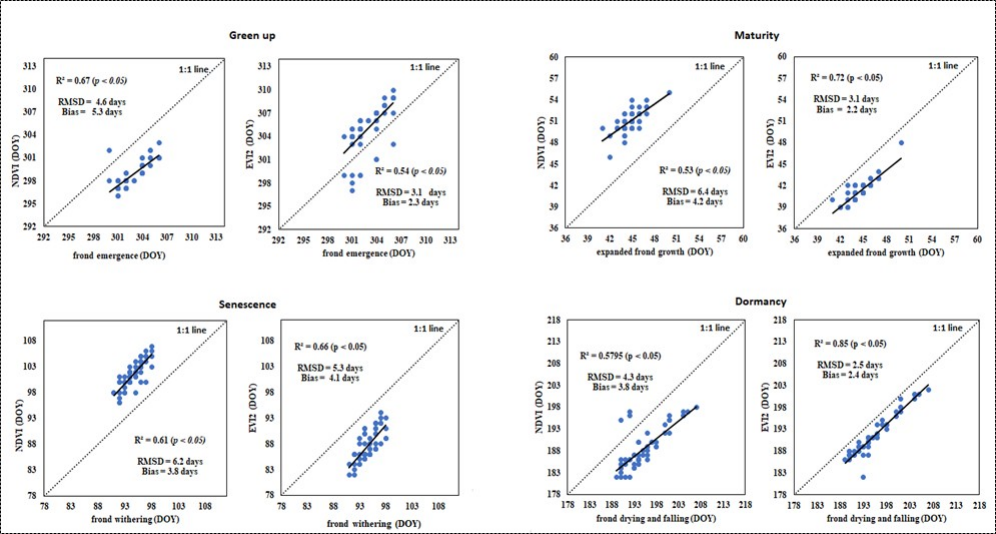
Statistical comparison between satellite-derived bracken fern phenological dates and ground observed phenology.

Bracken fern phenological metrics estimated using NDVI and EVI2 generally showed a good agreement with phenological dates from ground observations. Both EVI2 and NDVI phenological metrics show significant linear relationships (*p < 0*.*05*) with bracken fern ground observed phenological events with varrying correlations across all phenological stages. To provide a more comprehensive and quantitative assessment, Fig 5 shows scatter plots illustrating the statistical agreement between satellite-derived phenological metrics and ground observed transitional dates. The coefficients of determination for both NDVI and EVI2 phenological retrievals indicated a correlation with ground observed onset dates, with R^2^ values ranging from 0.53–0.85. The dormancy_EVI2_ phenological retrievals recorded the highest correlation (R^2^ = 0.85) with the ground observed frond drying and falling dates. The relationship between EVI2 retrieved dormancy onset dates and the ground observed bracken fern frond drying and falling showed a very strong correspondence for more than 75% of the pixels across the study site. The maturity _EVI2_ also reported a strong correspondence (R^2^ = 0. 72) with ground observed bracken fern expanded frond growth.

The RMSDs statistical values between satellite-based phenological retrievals and ground observed phenological transitional dates ranged from 2.5 to 6.4 days across the four bracken fern phenological stages. The RMSDs between NDVI and corresponding ground transitional dates were modestly higher (approximately one week) for senescence and dormancy phenological stages, while the EVI2 maturity and dormancy had the lowest RMSDs of 3.1 and 2.4 days respectively. The RMSDs between NDVI and ground recorded phenological dates were also higher (approximately one week) for maturity, senescence and dormancy while the green up phenological stage showed the lowest RMSD value of 4.6 days. The bias between satellite-based phenological retrievals and ground observed phenological events appeared to be very low as they ranged from 2.2 to 5.3 days. The largest bias (5.3 days) was recorded between green up _NDVI_ bracken fern frond withering, while the lowest bias (2.2 days) was reported between maturity _EVI2_ and bracken fern frond emergence. Generally, the EVI2 phenological retrievals corresponded more with bracken fern ground observed phenological events compared to NDVI phenological retrievals as shown by higher EVI2 correlation coefficients and lower RMSDs and Biases. Overall, the satellite-based bracken fern phenological estimates matched moderately well with the ground observed phenological events.

## 4. Discussion

### 4.1 The role of remotely sensed data in characterizing bracken fern phenology

The current study characterized the phenological cycle of bracken fern invasive species using NDVI and EVI2 time series data derived from the Sentinel-2 MSI sensor. The satellite-based phenological retrievals were compared with bracken fern ground observed phenological events. The Sentinel-2 sensor proved to be a reliable data source that could assist in improving the understanding of bracken fern phenological cycles, an aspect that could lead to better management of rangelands that are infected by the fern. Corresponding to our findings in this study, a plethora of scientific studies have also reported the capability of Sentinel-2 data 0.in extracting the phenological cycles of vegetation at various scales [34, 69–71]. Since Drakensberg is not prone to high cloud coverage, the sensor’s revisit time was sufficient to adequately capture the phenological changes of bracken fern. However, slight cloud coverage issues were experienced during the bracken fern maturity stage which coincided with the peak of the summer season. This could explain lower correlations between NDVI and bracken fern ground observed phenological events during the bracken fern maturity phenological stage.

The bracken fern phenological metrics estimated using the three models in the TIMESAT program were comparable across the four bracken fern phenological stages. The shape of the fitted NDVI and EVI2 curves in Fig 4 shows a possible quick response to precipitation, followed by a slow decay as bracken fern fronds withered. Corresponding with findings reported by Eklundh and Jönsson [72], our study established that the DL and AG models produced phenological curves that were subsequently used to estimate bracken fern phenological metrics that were more correlated to ground observed phenological events as compared to the SG model. Similarly, the works of Cai, Jönsson [73] also concluded that the AG and DL models produced a more robust and accurate description of the phenological cycles of vegetation compared to the other methods tested in their study including the SG model. Although the AG and DL produced similar bracken fern phenological curves, it can be noted that the AG well adapts and performs better during vegetation indices peaks as compared to the DL model. The works of Tan, Morisette [34] reported that the AG is less affected by noise and has a great advantage if the time series data has missing data or if the satellite data is poor quality due to sensor calibration errors. SG is mostly affected by atmospheric impurities and subsequently produces erroneous phenological metrics especially during the peak of the season where the data is characterized by clouds. Results from our study corresponded with previous findings by Li and Liu [74] and Khobkhun, Prayote [75] who reported that the SG model works well for data that is unaffected by noise caused by atmospheric contamination. Generally, our study demonstrated that the three models in TIMESAT perform good and they can reduce noise, reconstruct, and fit time series data for the estimation of bracken phenological metrics. However, the tuning of parameters is essential in the extraction of phenological metrics using these three models. The inappropriate selection of parameters may lead to uncertainty and bias in phenological trends produced by data smoothing models. The tuning of parameters in phenological metrics extraction was also raised in literature by Stanimirova, Cai [76] who highlighted that the differences in tuning parameters such as the use of 10% or 15% of seasonal amplitude as a benchmark threshold to ascertain the phenological metrics will yield different results.

### 4.2 Comparisons with ground observed phenological events

The validation of phenology metrics is essential for the evaluation of satellite sensors’ performance in estimating LSP. However, previous research studies have shown that the validation of remote sensing products is a huge challenge in LSP investigations [77–79]. Overall, the EVI2 performed better in estimating bracken fern phenological metrics that were correlated to ground observed phenological events. EVI2 proved to produce better estimates that are comparable to bracken fern ground observations during the maturity, senescence and dormancy phenological stages. Corresponding with our findings, Peng, Wang [80] noted that EVI2 significantly improves linearity with biophysical vegetation properties and reduces saturation effects found in densely vegetated surfaces, a challenge which is commonly encountered when using NDVI. Similarly, Zhang, Jayavelu [37] concluded that EVI2 is the better choice for detecting phenology than NDVI because EVI2 phenological retrievals were in close agreement with PhenoCam observations. The performance of EVI2 could also be attributed to its resistance to soil background effects which normally causes artificial increase in NDVI as reported by Rocha and Shaver [60]. On the other hand, NDVI outperformed EVI2 in retrieving the bracken fern green up onset. The performance of NDVI in estimating the onset of bracken fern green up could be attributed to its ability to reduce topographic effects [81] and illumination conditions [82] much better as compared to the EVI2.

The NDVI showed poor correlation with ground observation during the maturity phenological stage, while the EVI2 retrievals performed well during the maturity stage. As the bracken fern fronds increased in size and the canopy expanded, NDVI tends to saturate and become less efficient in extracting phenological metrics during the maturity period. The differences in phenological retrievals between NDVI and EVI2 probably originated from various resistance levels to noise and sensitivities to spectral signal at different bracken fern stage of the growing season. The NDVI’s poor performance in estimating the bracken fern maturity could probably be related to its loss of sensitivity when vegetation canopy’s leaf area index reaches a maximum as reported by Davi, Soudani [83] and Soudani, François [84]. Our results were consistent with Zuo, Liu [85] and Zhao, Yang [86] who reported that NDVI has more ability to track weak spectral signals in the early and end of vegetation growth season and tends to saturate at dense vegetation. Findings from the current study are consistent with previous work by Pettorelli, Vik [87] who noted that false highs occur when high NDVI values are considered to give better estimations than low values, and the bias tends to break the assumptions of many standard statistical methods. The rapid changes of NDVI during the bracken fern maturity period makes it complex for the determination of the phenological metrics. A study by Tan, Morisette [33] confirmed that vegetation normally changes quickly during green up and maturity, making it difficult to accurately detect changes in NDVI fluctuations.

### 4.3 Implications to the control and management of bracken fern

Challenges in controlling the encroachment of bracken fern into areas of ecological importance due to inappropriate timing, have been widely reported in literature [6, 13, 88, 89]. Therefore, the accurate estimation of bracken fern phenological transition times will help in the appropriate timing control measures and efforts of controlling the invasive fern for better management of the rangelands. Furthermore, the information on bracken fern’s phenology is vital in understanding the major drivers of its population dynamics and patterns of invasion. The effective management of rangelands requires continuous data sources that track the changes in various vegetation species that are within a landscape. Dawson, Jackson [90] highlighted that an effective conservation response must be broadly coordinated and informed by a range of scientific approaches with diverse data sources. The free availability of high spatial and temporal resolution data sets such as Sentinel-2 enables rangeland managers to continuously monitor the changes that occur within areas of their jurisdiction.

The debate with regards to the most suitable methods of controlling the spread of bracken fern has received much attention in the literature [13, 91, 92]. Findings from the current study suggest that the Sentinel-2 data is an invaluable tool that can be used as a foundation for decision-making particularly in controlling the spread of bracken fern in ecologically sensitive areas. The current study suggests that the development of bracken fern spores at the beginning of the senescence phase can be timely controlled using chemical measures such as spraying with asulum before they disperse. The application of chemicals on bracken fern during the senescence period significantly reduces the number of fronds that will be produced the following season [24]. Asulum does not affect in the year of application but kills almost all the buds on the rhizome which leads to less production of fronds in the following growing season. The mechanical control methods would probably be suitable during the green up phase when the bracken frond biomass is still low.

## 5. Conclusions and future works

The current study focused on characterizing the phenological cycle of bracken fern using NDVI and EVI2 time series data derived from the Sentinel-2 sensor. The satellite-based phenological retrievals showed a good correlation with bracken fern ground observed phenological events, making remote sensing technology a potential tool for effective bracken fern management. Inter-comparisons between NDVI and EVI2 based phenological metrics revealed that the two vegetation indices differ in their ability to track the phenological developments of bracken fern during its growing season. EVI2 is more suitable for retrieving LSP metrics than NDVI as it produced phenological metrics that were more related to bracken fern ground phenological events. The AG and DL models produced the best fits that optimally described the phenological profile of bracken fern. Through this study, remote sensing has been demonstrated to be an invaluable data source that can be used by conservationists, ecologists and rangeland managers in controlling and managing bracken-infested rangelands. The key phenology dates derived from the Sentinel-2 time series are relevant in informing stakeholders and policymakers on designing effective management strategies for controlling bracken fern invasion. After the successful characterization of bracken fern phenological cycle in the Drakensberg, the bracken fern phenological data may be used to improve the management of rangelands.
